# Culex Y Virus: A Native Virus of Culex Species Characterized In Vivo

**DOI:** 10.3390/v15010235

**Published:** 2023-01-14

**Authors:** Mareike Heinig-Hartberger, Fanny Hellhammer, David D. J. A. Zöller, Susann Dornbusch, Stella Bergmann, Katerina Vocadlova, Sandra Junglen, Michael Stern, Kwang-Zin Lee, Stefanie C. Becker

**Affiliations:** 1 Institute for Parasitology, University of Veterinary Medicine Hannover, Buenteweg 17, 30559 Hannover, Germany; 2 Research Center for Emerging Infections and Zoonoses, University of Veterinary Medicine Hannover, Buenteweg 17, 30559 Hannover, Germany; 3 Institute for Physiology and Cell Biology, University of Veterinary Medicine Hannover, Bischofsholer Damm 15, 30173 Hannover, Germany; 4 Fraunhofer Institute for Molecular Biology and Applied Ecology (IME), Ohlebergsweg 12, 35392 Giessen, Germany; 5 Institute of Virology, Charité Universitätsmedizin Berlin, Corporate Member of Free University Berlin, Humboldt-University Berlin, and Berlin Institute of Health, Chariteplatz 1, 10117 Berlin, Germany

**Keywords:** insect-specific virus, CYV, transmission, host-specificity, fertility

## Abstract

Mosquitoes are vectors of various pathogens that cause diseases in humans and animals. To prevent the outbreak of mosquito-borne diseases, it is essential to control vector populations, as treatment or vaccination for mosquito-borne diseases are often unavailable. Insect-specific viruses (ISVs) have previously been described as being potentially helpful against arboviral disease outbreaks. In this study, we present the first in vivo characterization of the ISV Culex Y virus (CYV). CYV was first isolated from free-living *Culex pipiens* mosquitoes in 2010; then, it was found in several mosquito cell lines in a further study in 2018. For mammalian cells, we were able to confirm that CYV does not replicate as it was previously described. Additionally, we found that CYV does not replicate in honey bees or locusts. However, we detected replication in the *Culex pipiens* biotype *molestus*, *Aedes albopictus,* and *Drosophila melanogaster*, thus indicating dipteran specificity. We detected significantly higher mortality in *Culex pipiens* biotype *molestus* males and *Drosophila melanogaster*, but not in *Aedes albopictus* and female *Culex pipiens* biotype *molestus*. CYV could not be transmitted transovarially to offspring, but we detected venereal transmission as well as CYV in mosquitos’ saliva, indicating that an oral route of infection would also be possible. CYV’s dipteran specificity, transmission routes, and killing effect with respect to *Culex* males may be used as powerful tools with which to destabilize arbovirus vector populations in the future.

## 1. Introduction

Mosquitoes are widespread vectors of various pathogens. In particular, mosquito-borne arboviruses such as dengue virus (DENV), West Nile virus (WNV), Sindbis virus (SINV), and chikungunya virus (CHIKV), to name a few, regularly lead to disease outbreaks in human populations and livestock worldwide [1]. Various factors such as climate change, tourism, and urbanization have led to the spread of mosquito species into new regions and increased incidences of mosquito-borne diseases in recent decades [2]. In addition to invasive mosquito species, resident species can also become vectors for introduced pathogens due to changes in environmental conditions [3]. Since neither effective vaccines nor drug treatment are available against most arboviruses, the common strategy is the prevention of infections by reducing vector populations, for example, through the massive use of insecticides. However, the use of insecticides bears many disadvantages, including a high risk of insecticide resistance in mosquito populations and harmful effects on the environment and other exposed organisms. Furthermore, repellents are often used to minimize the exposure of susceptible hosts to infected vectors. The disadvantages of repellents, besides the fact that repellents are not 100% effective, include the reduced sensitivity of virus-infected mosquitoes to repellents, which has been described, e.g., for SINV-infected *Aedes aegypti* mosquitoes [4]. Thus, new strategies for reducing arbovirus transmission are needed.

One strategy that has gained increasing attention in the past years is the use of insect-specific viruses or entomopathogenic bacteria and fungi to control vector populations. Insect-specific viruses (ISVs) are viruses from a variety of virus families that can only replicate in insect/arthropod hosts and, therefore, not in vertebrate cells [5]. They have been found in various arthropod species, sometimes due to a specific pathogenicity in their arthropod host, especially in insects cultivated for food or feed [6]. On the other hand, in many cases ISVs have been found incidentally in surveillance studies by screening hematophagous arthropods [5,7,8]. ISVs such as Nhumirim virus (NHUV), Culex flavivirus (CxFV), Palm Creek virus (PCV), Negev virus (NEGV), or cell-fusing agent virus (CFAV) have been described to affect the viral replication and transmission of arboviruses such as WNV [9,10,11], CHIKV [12], or Zika virus (ZIKV) [13] in vitro and in vivo when co-infected. By potentially affecting the vector’s competence or survival and fecundity, ISVs could be a useful tool for vector and virus transmission control. ISVs have also been used as platforms for diagnostic kits or vaccine candidates, e.g., for CHIKV [14]. Mosquito-derived ISVs were found in established cell lines persistently infected, for example, with CFAV, the first described ISV [15], in Aag2 cells (*Aedes aegypti* cells) as well as in laboratory mosquito colonies [10], but also in free-living mosquitoes [16,17,18]. To understand and exploit the potential of ISVs, more information on the pathogenicity, transmission, and spread of these viruses is needed. In 2010, a member of the *Birnaviridae* family, the Culex Y virus (CYV), was isolated from free-living *Culex (Cx.) pipiens* mosquitoes [17]. The *Birnaviridae* family includes four genera, with three genera (Aquabirnaviruses, Avibirnaviruses, and Blosnaviruses) infecting vertebrates excluding mammals, and a fourth genus, the Entmobirnaviruses, that infects insects [19]. In Germany, the native species *Cx. pipiens* is considered an important vector of WNV [20,21]. This mosquito species forms the so-called *Cx. pipiens* complex, which comprises several biotypes. The biotypes within this complex are morphologically indistinguishable and differ in terms of their essential ecological and behavioral characteristics; however, they can hybridize, thus significantly expanding their host range [22].

To expand our knowledge regarding the interaction of CYV with the *Cx. pipiens* biotype *molestus* mosquitoes, we characterized the virus’s replication in vivo, mosquito survival after infection, and the possible transmission routes of CYV. Furthermore, we analyzed the host specificity of this virus by conducting in vitro experiments with mammalian cells and in vivo experiments with the *Cx. pipiens* biotypes *molestus* (mosquitoes), *Aedes (Ae.) albopictus* (mosquitoes), *Drosophila (D.) melanogaster* (fruit flies), *Apis (A.) mellifera* (honey bees), and *Locusta (L.) migratoria* (locusts). All in all, we propose that CYV might be a candidate for new vector control strategies in the future.

## 2. Materials and Methods

### 2.1. Insects, Climate Chambers, and Rearing Conditions

The *Cx. pipiens* biotype *molestus* (Forskal, 1775) colony was established in our laboratory in Hannover, Germany, in 2016, and was derived from blood-fed gravid females collected in 2012 in Wendland (W strain), Germany, and from egg rafts collected in 2013 in Langenlehsten (LL strain), Germany [23]. In addition, a hybrid strain from the W and LL strains was established in 2019 and also used for the experiments. The *Cx. pipiens* biotype *molestus* colonies were maintained in climate chambers at 26 °C (±1 °C), a relative humidity of 45–75%, and a 16:8 (light:dark) hr photoperiod with 1 h twilight periods at dusk and dawn. Our *Cx. pipiens* biotype *molestus* mosquito colony was naturally infected with *Wolbachia* bacteria. They tested negative for Alphaviruses, Orthobunyaviruses and Flaviviruses (data not shown); the primer sets and thermal conditions employed for amplification have already been published [24,25,26,27]. An *Ae. albopictus* (Skuse, 1894) colony from Nice, France, was provided by Infravec2 (Laboratory of Prof. Failloux), Nice, France, and was maintained at 28 °C (±1 °C), a relative humidity of 60–75%, and a 16:8 (light:dark) hr photoperiod with 1 h twilight periods at dusk and dawn. Mosquito larvae were reared in plastic basins or bowls filled with dechlorinated tap water and fed with TetraPleco fish food tablets (Tetra Werke, Melle, Germany) until pupation and hatching. Adult mosquitoes were housed in cages (BugDorm-1, 30 cm × 30 cm × 30 cm; Bioquip, Compton, CA, USA) and fed ad libitum with 8% fructose solution. As a dietary supplement, 0.5 g/L of 4-aminobenzoic acid (PABA) was added to the fructose solution. Adult female mosquitoes were fed with dog blood once a week.

Mosquitoes used for the experiments were between two and seven days old. All experiments with mosquitoes were performed in rearing cages (BugDorm-1) in a climate chamber at 26 °C (±1 °C), relative humidity of 45–75%, and a 16:8 (light:dark) hr photoperiod with 1 h twilight periods at dusk and dawn.

Flies of the *D. melanogaster* yellow white (YW) strain from Bloomington Drosophila Stock Center were reared in drosophila cultivation tubes (ø 50 mm; Carl Roth GmbH + Co. KG, Karlsruhe, Germany) filled with approx. 2 cm of corn flour meal agar (35 g of corn flour (Alnatura, Darmstadt, Germany), 8.5 g of dry yeast (Alnatura, Darmstadt, Germany), 23.5 g of glucose (Carl Roth GmbH + Co. KG, Karlsruhe, Germany), 5 g of sugar beet syrup (Bauckhof, Germany), 0.04 g of Nipagin (Carl Roth GmbH + Co. KG, Karlsruhe, Germany), 4 g of agar (Carl Roth GmbH + Co. KG, Karlsruhe, Germany), 1.625 mL of propionic acid (Carl Roth GmbH + Co. KG, Karlsruhe, Germany), and 400 mL of tap water) to provide food and moisture. *D. melanogaster* specimens were held at 24 °C (±1 °C) and 45–55% RH in constant darkness, i.e., without a light:dark cycle.

Locusts were reared in a 12:12 cycle (light:dark) at 30 °C in crowded culture in the facilities of the Institute of Physiology and Cell Biology of the University of Veterinary Medicine Hannover. The animals were fed ad libitum with fresh wheatgrass and wheat bran. Adult locusts were offered tubes filled with moist sand and water-soaked vermiculite for egg laying. Egg tubes were incubated at 30 °C until eclosion.

Honey bees were collected from two *A. mellifera* colonies from the apiary of the Justus-Liebig University (Giessen, Germany, 50°34′06.72″ N 8°40′19.7″ E) in the second half of August 2021. A piece of capped brood comb with late-stage pupae was incubated at 35 °C and 70% relative humidity. The newly emerged bees were transferred to a conical plastic box (18 cm × 18 cm × 7.9 cm) and fed sugar solution (1:1 sucrose and water) ad libitum.

### 2.2. Cell Culture

Mammalian cells used for infection experiments were BHK-21 (hamster kidney cells; CCVL L 0179) and A549 (human lung epithelial cells; ATCC^®^ CCL-185™). Cells were cultured in Dulbecco’s Modified Eagle Medium (DMEM; Thermo Scientific Inc., Waltham, MA, USA) (A549) or Minimum Essential Medium (MEM; Capricorn Scientific, Ebsdorfergrund, Germany) (BHK-21), both supplemented with (1%) penicillin–streptomycin (100 units/mL) (PAN-Biotech GmbH, Aidenbach, Germany), (1%) L-glutamine (200 mM) (PAN-Biotech GmbH, Aidenbach, Germany), and 10% FBS (A549) or 5% FBS (BHK-21) (Capricorn Scientific, Ebsdorfergrund, Germany) at 37 °C and 5% CO_2_. Cell cultures were split twice weekly to prevent cells from reaching full confluence. To detach the cells from the bottom of the flask, cells were treated with trypsin.

### 2.3. Homogenization, RNA Extraction, RT-PCR, qRT-PCR, and CYV Standard Preparation

All insect samples were homogenized before further analyses. For this purpose, insect samples were transferred in groups or individually, depending on the assay, to reaction tubes containing 500 µL of Schneider’s Drosophila Medium (PAN-Biotech GmbH, Aidenbach, Germany) or 1 mL of nuclease-free water (bees) containing two 5 mm steel balls (Isometall, Pleidelsheim, Germany) and were homogenized using the TissueLyser II (Qiagen, Hilden, Germany) at 30 Hz for 30 s or 25 Hz for 3 min (bees). Samples were then centrifuged at full speed (13,300× *g*) for 1 min, and the homogenate was frozen at −18 °C until further processing. RNA extraction was performed using the QIAamp Viral RNA Mini Kit (Qiagen, Hilden, Germany), according to the manufacturer’s instructions.

To quantify CYV RNA copy numbers, primers OSM_318 (5′ CCAGAGAATGTGAAGAGA 3′), OSM_319 (5′ CGTTGTTAAGGAAGACTC 3′), and a probe OSM_320 (FAM-CACAAGAGGATACACAAGCAGCG-BHQ1) were used. The Luna Probe One-Step RT-qPCR Kit (New England Biolabs, Ipswich, MA, USA), was used for all samples with 9 µL master mix and 1 µL template in an AriaMx Real-time PCR System (Agilent Technologies, Santa Clara, CA, USA). The thermal program consisted of the application of 55 °C for 10 min, followed by 1 min at 95 °C and 40 cycles of 10 s at 95 °C and 30 s at 54 °C (annealing). The resulting fragment was 114 bp long.

A PCR standard was prepared using the SuperScript III One-Step RT-PCR Platinum Taq DNA Polymerase Kit (Thermo Fisher Scientific Inc., Waltham, MA, USA) according to the manufacturer’s instructions, thus reducing the final volume from 50 μL to 25 μL, including 1 µL template. A fragment length of 414 bp was amplified using primers OSM_321 (5′ GGCAATGTTCGGGCTCATC 3′) and OSM_322 (5′ CGACAGTGCAGGAGTAGGGG 3′). The cycler program was set to 1 min at 60 °C and 45 min at 50 °C, followed by 2 min 95 °C for reverse transcriptase. This was followed by 40 cycles of DNA denaturation at 94 °C (15 s), annealing at 59 °C (30 s), and elongation at 68 °C (30 s). A final elongation of 7 min was carried out. The resulting PCR product was then purified using the NucleoSpin Gel and PCR Clean-up Kit (Macherey and Nagel, Düren, Germany) according to their instructions. For transcription, a T7 promoter was added to the previous purified PCR product. For this purpose, a T7 promoter was added to the standard OSM_321 forward primer, OSB_728 (5′ TAATACGACTCACTATAGGGGGCAATGTTC 3′). The RNA concentration was measured, and the RNA copy number/mL was calculated. The detection limit for our standard was 1 × 10^4^ RNA copies, which had Ct values between 33 and 35; thus, measurements over Ct value 35 were excluded in further analyses.

### 2.4. Injection

CO_2_-anesthetized adult mosquitoes were injected in the lateral metathorax [28,29] using a Nanoject II Auto-Nanoliter injector (Drummond Scientific Company, Broomall, PA, USA). The injection solution used was either the CYV isolate of Franzke et al. (2018) [30] (first isolated in 2010 by Marklewitz et al. [17]) (virus-infected groups) or Schneider’s Drosophila Medium (control groups). The injection rate was set at 23 nL per second. All experiments were performed in at least three individual replicates; bees’ survival was replicated in two independent replicates. An RNA standard was used to quantify CYV because the virus did not show a cytopathic effect in any of the cell cultures tested. Therefore, we used the RNA copy numbers to standardize our experiments and ensure reproducibility. Mosquitoes were then injected with 46 nL.

### 2.5. In Vivo Growth Kinetics in Cx. pipiens Biotype Molestus

In vivo kinetics of viral growth in *Cx. pipiens* biotype *molestus* were monitored by measuring viral RNA copy numbers at the following days post-injection: zero, three, seven, and fourteen. Mosquitoes from all three described strains (W, LL, and hybrid) were sampled in equal numbers of males and females. To study viral growth and dissemination, mosquitoes were injected with 46 nL of CYV (approximately 3.32 × 10^5^ RNA copies) and RNA copies were measured by qRT-PCR using the CYV standard described in Section 2.3. In total, 72 males and 72 females were dissected starting on the day of injection, wherein mosquitoes were dissected within two hours after injection. On each dissection day, six males and six females (two individuals per sex per mosquito strain) were anesthetized with CO_2_ and immobilized by removing legs and wings. The immobilized mosquitoes were then rinsed with 70% ethanol to sterilize their exoskeletons and remove static charge from the mosquitoes; then, they were placed on a microscope slide in 50 μL of 1× phosphate-buffered saline (PBS; pH 7), which provides necessary physiological conditions and prevents contamination of the hemolymph. The salivary glands (females only), ovaries or testes, intestines, heads, and carcasses were dissected and washed three times in 200 µL 1× PBS and then pooled in 1.5 mL reaction tubes containing 500 µL Schneider’s Drosophila Medium and two 3 mm steel balls and stored at −18 °C until further processing.

### 2.6. Transmission of CYV within Cx. pipiens Biotype Molestus Populations

#### 2.6.1. Vertical Transmission

To test vertical transmission of CYV within mosquito populations, a total of 126 virgin males and 324 virgin females were infected with CYV by injection and a control group, containing 108 virgin females and 42 virgin males, was injected with Schneider’s Drosophila Medium. To ensure virginity on the day of injection and prior to assembly, pupae were placed in individual *Drosophila* tubes until hatching. After injection, males and females were placed in BugDorm-1 cages and fed ad libitum with an 8% fructose solution under conditions described in Section 2.1. The progeny (adults of the F1 generation) from autogenous oviposition of CYV-infected mosquitoes were reared and tested for CYV.

#### 2.6.2. Venereal Transmission

Virgin mosquitoes were injected and then incubated again separately sexed for five days. On the fifth day, CYV-infected mosquitoes were joined with virgin CYV-naïve mosquitoes of the opposite sex. After seven days, the surviving individuals, consisting of a total of 168 CYV-injected females, 127 CYV-injected males, 70 CYV-naïve females, and 71 CYV-naïve males, were pooled into a maximum of twelve individuals per pool and sexed and processed as described for qRT-PCR analyses. The minimum infection rate (MIR) was calculated for this experiment as described by Pettersson et al. (2014) [31], since all samples were tested in pools. The number of individuals in each pool was different; thus, RNA copy number per animal was calculated for statistical analyses.

#### 2.6.3. Saliva Assay

For the oral transmission experiments *Cx. pipiens* biotype *molestus* mosquitoes were injected with CYV two to three days after hatching, as described earlier, and caged until five days post-infection (dpi) to ensure viral growth. We examined saliva from 30 CYV-injected males and 30 CYV-injected females. Saliva was obtained by forced salivation of male and female mosquitoes via removal of wings and legs and placing the proboscises in 10 µL of 1× PBS using a pipette tip. Mosquitoes were allowed to feed for 1.5 h. Saliva and mosquito bodies were then tested for CYV by qRT-PCR.

#### 2.6.4. Oral Infection

Furthermore, we performed viral growth kinetic experiments on orally CYV-infected mosquitoes with a total number of 144 CYV-infected mosquitoes comprising 72 males and 72 females. An 8% fructose solution containing 35 µL of red ink as a dye and 5 µL of CYV containing approximately 3 × 10^6^ RNA copies was prepared. Feeding was performed in tubes in groups containing a maximum of 12 animals each. The mosquitoes were allowed to feed on the fructose solution containing CYV via cotton swabs (200 µL per cotton swab) for 180 min. Animals with distinctly red-colored abdomens were used for growth kinetics experiments, as described in Section 2.5.

### 2.7. Oviposition and Progeny Outcome of CYV-Injected Cx. pipiens Biotype Molestus

All autogenous egg rafts laid by CYV-injected females described in Section 2.6.1 were photographed using a Moticam 5 camera (Motic, Barcelona, Spain) and the Motic Images Plus 2.0 software (Motic, Barcelona, Spain), and the quantities of egg rafts and eggs were counted using ImageJ 1.53c software (Wayne Rasband, National Institutes of Health, Bethesda, MD, USA). Then, the egg rafts were kept in tanks filled with tap water, and hatched larvae were fed 70–80 mg of a TetraPleco fish-food tablet daily. From the late L_2_ instar to the early L_3_ instar, 100 larvae were kept at a constant density of 4 cm^3^/larvae to prevent the crowding factor from affecting development. Resulting pupae were counted and hatched mosquitoes were frozen at −18 °C.

#### 2.7.1. F1 Generation—Progeny of CYV-Infected Females of *Cx. pipiens* Biotype *Molestus*

Wings from 295 frozen female offspring and 286 frozen male offspring from CYV-infected mosquitoes and from 102 female and 97 male offspring from medium-infected mosquitoes were severed near the thorax with precision forceps and wing length (WL) and wing area (WA) were measured. The detached wings were placed on a white sheet of paper with a scalable grid for size reference. A glass slide was carefully placed on the wings to straighten them. Wings were photographed using a Moticam 2.0 camera on a binocular microscope at 10× magnification and measured using ImageJ 1.53c. Wing length was measured using the linear distance between the distal end of the alula and the tip of the third radial vein at the apical margin. Wing area was defined as the area on the upper surface excluding the fringe. The proximal end of the area was defined by a vertical line connecting the end of the fringe and the posterior margin of the alula [32]. WL and WA were measured for the left and right wings and the final WL was determined as the mean of the size of the right (WLR) and left (WLL) wings [(WLR + WLL)/2]. The same was performed for the WA [(WAR + WAL)/2].

#### 2.7.2. Survival Assays

We monitored the survival of 96 CYV-injected *Cx. pipiens* biotype *molestus* males and 96 CYV-injected *Cx. pipiens* biotype *molestus* females. A control group injected with Schneider’s Drosophila Medium that comprised the same number of mosquitoes was also included in each replicate. Mosquitoes were injected as described in Section 2.4, and observation began 24 h post-injection to exclude dead mosquitoes due to injection. We then monitored mosquito survival daily over a 14-day period and removed any dead individuals from the cages. At least 10% of dead individuals were tested for CYV to validate infection status.

### 2.8. In Vitro CYV Infection of Mammalian Cell Cultures

Mammalian cells (BHK-21 and A549) were seeded in 12-well plates (Sarstedt AG & Co. KG, Nümbrecht, Germany) at 0.1 × 10^6^ cells per well with 500 µL of the appropriate cell culture medium and 10% FBS and allowed to grow to approximately 80% confluence. Approximately 3 × 10^7^ CYV RNA copies were added to 500 µL of Schneider’s Drosophila Medium and used for infection. Mammalian cells were inoculated at 37 °C and 5% CO_2_ for one hour, with plates gently shaken every 15 min. As stated above (2.4), CYV did not induce any CPE in cells, thus hindering a regular plaque-forming unit assay; therefore, we used RNA copies of the virus to standardize our experiments. After inoculation, the inoculum was removed, and the cells were washed three times with 500 µL 1× PBS (pH 7) and replenished with medium and FBS. Viral kinetics were monitored for seven days, with 200 µL of supernatant collected on day zero, day three, and day seven post-inoculation. Infection and negative controls were performed and treated in triplicate and in three independent experiments.

#### In Vivo CYV Infection of Other Insects

We further investigated the host specificity of CYV by injecting the virus into other insect species. For all infection experiments, each replicate included a virus-infected group and a Schneider’s Drosophila Medium control group. Individuals that died within 24 h post-injection were excluded from further analyses.

For this investigation, *Ae. albopictus* mosquitoes were injected with 46 nL of CYV (approximately 3.32 × 10^5^ RNA copies), as described in Section 2.4. A total of 144 mosquitoes consisting of 72 males and 72 females injected with CYV and the corresponding control groups were observed in survival experiments conducted as described in Section 2.7.2. Dead individuals were removed and tested for CYV via qRT-PCR. Viral growth was tested in pools of five males and five females per pool after CYV infection at days zero, one, seven, ten, fourteen, and twenty-one via qRT-PCR, and viral growth was determined.

*D. melanogaster* flies of a yellow white wildtype (YW) strain, as representatives of Brachycera, were injected with 18.4 nL of CYV (approximately 7.34 × 10^3^ RNA copies) or Drosophila Schneider’s Medium as control group, respectively. In total, 125 males and 125 females per virus and control group (three to six days old) were observed. For the experiments, two to three *D. melanogaster* tubes (ø 50 mm; Carl Roth GmbH + Co. KG, Karlsruhe, Germany) of strain YW were completely emptied. On the third day after hatching, the newly hatched individuals were placed on fresh drosophila cultivation tubes (ø 50 mm; Carl Roth GmbH + Co. KG, Karlsruhe, Germany) filled with approx. 2 cm of corn meal agar and left for three days at 24 °C (±1 °C) and 45–55% RH in constant darkness to allow the immune system of flies fully develop. After injection, 50 flies (25× males and 25× females) per group were placed in fresh *Drosophila* tubes containing cornmeal agar and maintained under the same conditions as *Cx. pipiens* biotype *molestus* mosquitoes. The flies were checked every day for survival, except on weekends. Dead flies were removed from the tubes and stored at −18 °C. After seven days post-infection, the flies were replaced in new *Drosophila* cultivation tubes to ensure that generations did not mix. We performed survival experiments over 14 days. Dead individuals were removed from the tubes and tested for CYV via qRT-PCR. For viral growth, fruit flies were injected as described and individual pools of five males and five females per pool were tested for CYV at day zero, one, five, ten, and fourteen after injection.

Specimens of *A. mellifera*, the honey bee, a member of the Hymenoptera order, were injected with 1 µL of CYV (approximately 8.17 × 10^5^ RNA copies) or Schneider’s Drosophila Medium. The bees were quickly anesthetized with CO_2_ and their thoraxes were injected using Nanoject II (Drummond Scientific Company, PA, USA). Subsequently, the bees were distributed into the plastic experimental boxes (11.5 cm × 8.5 cm × 8 cm) with a piece of wax, provided with sucrose solution (1:1) ad libitum, and kept under the conditions described above. A total of 29 bees were injected with CYV and 51 were injected with medium for survival experiments described in Section 2.7.2.

For the study of viral growth kinetics, 36 bees were injected as described above and samples were analyzed for CYV by qRT-PCR at four time points post-injection: zero, three, seven, and fourteen. Two live bees per treatment were frozen and stored at −20 °C until further analysis.

*L. migratoria*, a member of the order Orthoptera, was also injected with CYV. For each treatment group (Schneider’s Drosophila Medium as control and the CYV-injected group), 24 first-instar locusts were injected a few hours after eclosion [33] with a volume of 193.2 nL (3 × 64.4 nL) of either Drosophila Schneider’s Medium as control or CYV (approximately 4.98 × 10^5^ RNA copies). Locusts were kept in groups of six in *Drosophila* tubes filled up to 2 cm with agar to provide adequate humidity and were maintained at ambient conditions in the laboratory (temperature: 22–23 °C; humidity: 20–40%). The agar-filled tubes were replaced every second day, and the animals were fed with fresh or thawed wheatgrass daily. We performed survival experiments as described in Section 2.7.2, and dead individuals were tested for CYV via qRT-PCR.

### 2.9. Statistical Analyses

All statistical analyses were performed using Prism 9 Version 9.4.0 (San Diego, CA, USA). To determine the survival rates, Kaplan–Meier survival curves were generated and significance levels between curves were determined using the log-rank test.

Data were tested for normality using the Shapiro–Wilk test. Parametric analyses of more than two groups were conducted with a one-way ANOVA test or two-way ANOVA test, while non-parametric data were tested using the Kruskal–Wallis test. Individual testing of two groups was performed using the unpaired *t*-test with Welch’s correction for parametric data and the Mann–Whitney test for non-parametric data. Multiple *t*-tests were performed using Bonferroni correction. Results of tests were considered significant when *p* ≤ 0.05. Significance levels were displayed in four levels by different numbers of asterisks: *p* ≤ 0.05 is shown as *, *p* ≤ 0.01 is marked with **, *p* ≤ 0.001 with ***, and *p* ≤ 0.0001 is marked with ****.

## 3. Results

### 3.1. In Vivo Growth Kinetics in Cx. pipiens Biotype Molestus

**Figure 1:**
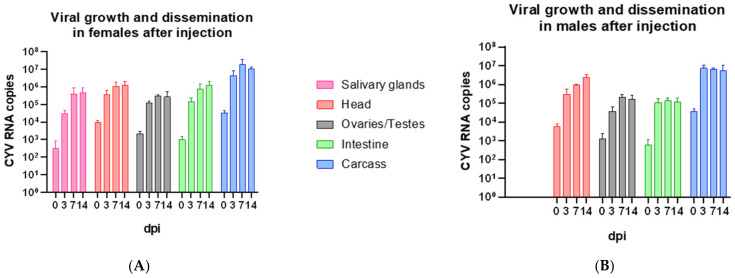
Growth kinetics of CYV after injection in *Cx. pipiens* biotype *molestus*. Mosquito dissection and preparation of different organs at zero dpi, three dpi, seven dpi, and fourteen dpi. Mean RNA copy numbers per organ per individual are displayed per dissection day with SD. (**A**) Viral growth in organs of 72 females; (**B**) viral growth in organs of 72 males. Pink—salivary glands; red—head; grey—ovaries/testes; green—intestine; blue—carcass; dpi = days post-infection.

To investigate viral replication and dissemination in vivo, the viral growth kinetics of CYV were examined. Accordingly, viral RNA was measured in the heads, carcasses, reproductive organs, intestines, and salivary glands (females only) at four time points after CYV injection (zero, three, seven, and fourteen dpi) in *Cx. pipiens* biotype *molestus* mosquitoes. In total, we dissected 72 males and 72 females divided into 3 independent replicates. The results show that within two hours after its injection into the thorax, all organs were flooded with CYV, although all organs were washed three times in 1× PBS. We obtained an increase in RNA copy numbers in all the tested organs of the females at day three and day seven post-injection (Figure 1A). Except for the ovaries and carcass, where a slight decrease in RNA copy numbers was observed, the RNA increase could still be measured on the fourteenth day post-injection. The kinetics of viral growth in the males after injection showed a similar pattern as that in the females, with an additional slight decrease in the intestine on the fourteenth day (Figure 1B). In almost all the analyzed samples, the number of RNA copies was slightly lower in the males compared to the females but did not differ significantly (for all organs, α > 0.05; Welch’s *t*-test) (Figure 1A,B). The lowest RNA copy number was measured in the intestine of the males with 7.15 × 10^2^ RNA copies on the day of injection. The highest RNA copy numbers were measured in the carcasses of both sexes at all sampling days, ranging from 3.28 × 10^4^ at day zero to 1.08 × 10^7^ at day fourteen in females and 3.70 × 10^4^ at day zero to 8.91 × 10^6^ at day fourteen in males. The lowest RNA copy numbers were found in the females’ salivary glands and the intestines of males and females on the day of injection. The mean RNA copy numbers of all the organs combined yielded a copy number of 4.44 × 10^4^ in males and 4.56 × 10^4^ in females on the day of injection. Compared with the RNA copies in the inoculum (3.32 × 10^5^ per injection), the measured RNA copies in the mosquitoes’ organs were lower after injection. On the fourteenth day, higher amounts of viral RNA were measured for all organs compared to the inoculum, namely, 1.16 × 10^7^ in males and 1.40 × 10^7^ in females. The data shown include measurements that passed the detection limit.

Statistical analysis of viral RNA copy numbers per organ and sex over four time points showed that, with the exception of the carcass, no significant differences in viral growth were detected (α > 0.05; Multiple *t*-test, Bonferroni-corrected). In the carcasses, significantly lower viral RNA copy numbers were detected in both sexes at day zero compared to day seven (males: *p* = 0.0177; Multiple *t*-test, Bonferroni-corrected; females: *p* = 0.0425; Multiple *t*-test, Bonferroni-corrected) and day fourteen in males (*p* = 0.0086; Multiple *t*-test, Bonferroni-corrected). In addition, a significant difference was observed between day zero and day three in males (*p* = 0.0141; Multiple *t*-test, Bonferroni-corrected) (Table 1).

### 3.2. CYV Transmission Experiments with Cx. pipiens Biotype Molestus

We investigated how CYV could be transmitted within a mosquito population. For this purpose, we tested different transmission routes in *Cx. pipiens* biotype *molestus* mosquitoes, namely, vertical, venereal, and oral transmission, in the following set of experiments.

#### 3.2.1. Vertical Transmission

To investigate whether the virus could be transmitted vertically, we first examined CYV replication in the reproductive organs of both sexes (Figure 1A,B). We found that CYV replicated in the reproductive organs of the males and females at nearly identical levels, ranging from 1.35 × 10^3^ and 2.16 × 10^3^ RNA copies in the testes and ovaries, respectively, on day zero to 1.67 × 10^5^ and 2.72 × 10^5^ on day fourteen. The peak viral RNA was measured on day seven in both sexes, with 3.03 × 10^5^ in the ovaries and 2.06 × 10^5^ in the testes.

Next, we tested the vertical transmission of the virus in the CYV-positive mosquitoes to their offspring. Therefore, 672 adult F1 generation mosquitoes were tested for viral RNA copies; consequently, it was revealed that none of the tested samples had tested positive. Therefore, it seems that the vertical transmission of CYV does not occur in *Cx. pipiens* biotype *molestus* mosquitoes.

#### 3.2.2. Venereal Transmission

**Figure 2:**
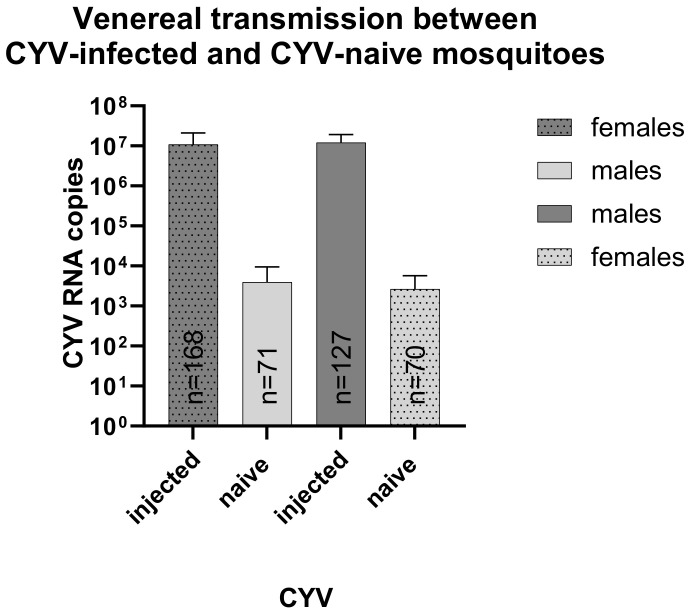
CYV measurement of previously CYV-naïve *Cx. pipiens* biotype *molestus* mosquitoes after mating with CYV-positive mating partners. Shown are mean viral RNA copies measured by qRT-PCR calculated for one mosquito (per pool) seven days post-mating, displayed with SD. Dark grey with dots: CYV-injected females (n = 168); dark grey—CYV-injected males (n = 127); light grey with dots: CYV-naïve females (n = 70); light grey—CYV-naïve males (n = 71).

Another potential route of viral spread within a population might be the venereal transmission of CYV from CYV-positive to CYV-naïve mosquitoes. The RNA copy numbers were analyzed per mosquito pool; in a second step, we calculated the RNA copy number per individual animal from these data to enable better comparability between pools (Figure 2). To test this, we mated CYV-positive males or females with their non-infected counterparts. We were able to detect CYV RNA in all the tested pools of the priorly CYV-naïve females and males. The samples were tested in pools, which resulted in a minimum infection rate (MIR) of 8.57% for the females (six pools) and 9.86% for the males (seven pools). The transmission occurred both from females to males and from males to females. We measured a mean RNA copy number of 1.09 × 10^7^ 12 days post-injection for the females, which was calculated per animal. Their male mating partners, who were originally CYV-naïve in this experiment, had a mean RNA copy number of 3.97 × 10^3^ per animal. Similar results were observed for the CYV-injected males 12 days after injection, with a mean RNA copy number per animal of 1.21 × 10^7^. An average of 2.65 × 10^3^ viral RNA copies were detected in their female, previously CYV-naïve mating partners.

#### 3.2.3. Oral Transmission

**Figure 3:**
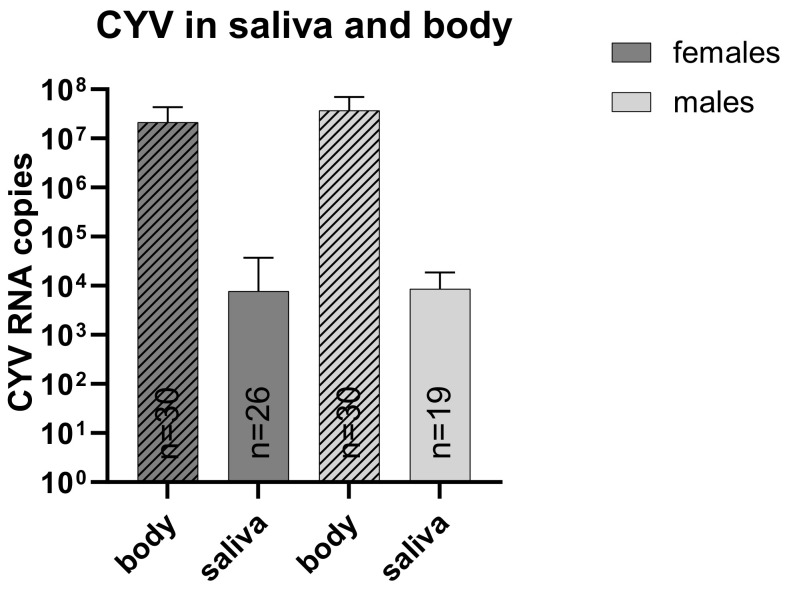
CYV measurement in saliva and carcasses five days after injection in *Cx. pipiens* biotype *molestus* mosquitoes after forced salivation. Shown are mean viral RNA copies measured by qRT-PCR per mosquito displayed with SD. Dark grey with lines—female bodies (n = 30); dark grey—female saliva (n = 26); light grey with lines—male bodies (n = 30); light grey—male saliva (n = 19).

To investigate whether CYV could be transmitted orally in the mosquito populations, first, we conducted a forced salivation assay with the CYV-injected mosquitoes and additionally performed feeding experiments to determine whether the virus could be ingested in the diet and replicate in the mosquitoes. Forced saliva testing confirmed that 100% of the CYV-injected mosquitoes were positive for CYV infection after injection. Regarding the females, 86.7% also had CYV-positive saliva, with a mean RNA copy number of 7.76 × 10^3^, while 63.3% of the males had CYV-positive saliva with a mean RNA copy number of 8.62 × 10^3^ in the male saliva (Figure 3). RNA copy numbers in the bodies of females and males were significantly higher (for both sexes: α < 0.0001; Mann–Whitney test) than in their respective saliva samples: 2.14 × 10^7^ (females) and 3.69 × 10^7^ (males).

**Figure 4:**
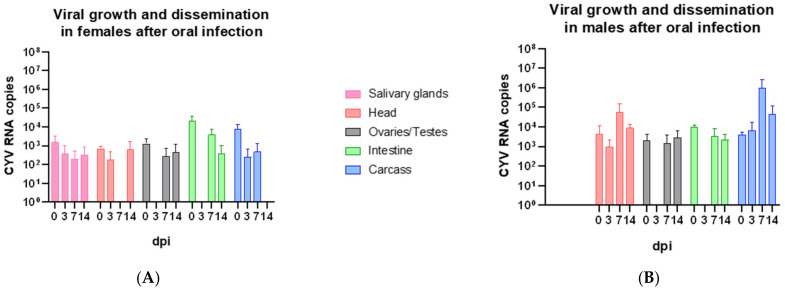
Growth kinetics of CYV in several organs after oral infection in *Cx. pipiens* biotype *molestus*. Mosquito dissection and preparation of different organs at zero dpi, three dpi, seven dpi, and fourteen dpi. Organs were washed three times in 1× PBS. RNA copy numbers per organ per individual are displayed per dissection day with SD. (**A**) Viral growth in females (n = 48); (**B**) Viral growth in males (n = 72). Pink—salivary glands, red—head, grey—ovaries/testes, green—intestine, and blue—body; dpi = days post-infection.

In a second step, we found that CYV can be taken up via a fructose solution and that the virus replicates after oral uptake in both sexes (Figure 4). All organ pools of fed males and 66.7% of the female pools were tested positive for CYV RNA after feeding. The RNA copies of CYV decreased in all organs of both sexes from the day of infection to day three post-infection, indicating the digestion of the initial inoculum. No virus was detected in the reproductive organs and intestines of the females and males on the third day after oral uptake. However, after seven days of infection, an increase in RNA copies was observed in all the organs except the female salivary glands and the head, in which no viral RNA was detected. Fourteen days after oral infection, no viral RNA copies were detected in the females’ carcasses and the highest in the males’ carcasses with 4.29 × 10^4^ RNA copies. The mean RNA copy numbers of all organs combined resulted in a copy number of 4.98 × 10^3^ in the males and 7.07 × 10^3^ in the females on the day of oral infection.

### 3.3. Survival

**Figure 5:**
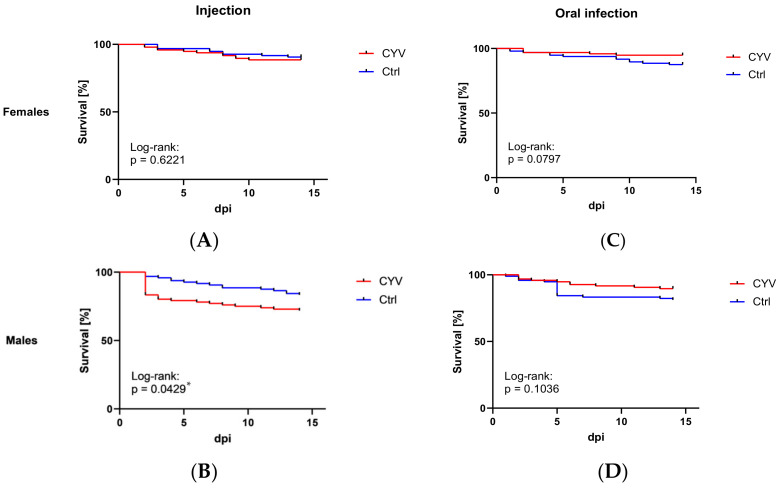
Survival curves of infected *Cx. pipiens* biotype *molestus* mosquitoes comparing CYV-infected and control mosquitoes per sex and infection route. The data represent the survival rates in percentages over days post-infection (dpi). (**A**) CYV-injected females vs. medium-injected females; Log-rank: *p* = 0.6221 (ns). (**B**) CYV-injected males vs. medium-injected males; Log-rank: *p* = 0.0429 (*). (**C**) CYV-fed males vs. medium-fed males; Log-rank: *p* = 0.1036 (ns). (**D**) CYV-fed females vs. medium-fed females; Log-rank: *p* = 0.0797 (ns). Red curve—CYV-infected mosquitoes; blue curve—control (Ctrl) mosquitoes.

To investigate whether CYV has a negative impact on mosquito survival, we monitored the CYV-infected mosquitoes in a survival assay for 14 consecutive days and counted the surviving individuals. The survival of the female mosquitoes was not significantly impaired by CYV infection in both the injected and the oral infection group (Figure 5A,C). In male mosquitoes, a significantly reduced survival rate was observed in the CYV-injected group, with only 72% surviving 14 days after infection (Figure 5B). No significant differences were observed in the orally infected males between the control and virus-infected groups (Figure 5D).

### 3.4. Impact on Reproduction

To investigate whether there could be effects on reproduction in terms of egg rafts, eggs, hatching, pupation and emergence rates, these traits were analyzed based on the viruses found in the reproductive organs. No significant differences were found between CYV- and medium-injected females and their offspring for all traits tested (Table 2). When egg rafts were counted, an average of 0.66 (±0.157) egg rafts per female were laid in the virus-injected group compared with 0.59 (±0.121) egg rafts in the medium-injected females. We also found a slightly higher number of eggs per female (26.3 ± 10.48) and per egg raft (39.2 ± 10.23) in the CYV-injected females compared to the control group (21.4 ± 9.97 and 35.6 ±9.73, respectively). Upon hatching from eggs, the progeny of the medium-injected mosquitoes had higher hatching (88.2% ±9.84%), pupation (97.3 ± 3.79%), and emergence (96% ±5.39%) rates than those of the virus-injected parental generation (hatching from eggs: 76.9% ±9.13%, pupation: 88.7% ±11.48%, and emergence: 82.8% ±16.15%). The number of female offspring that emerged was also higher in the control group (55.4 ± 4.15%) than in the virus group (49.3% ± 6.34%).

### 3.5. Impact on Progeny

**Figure 6:**
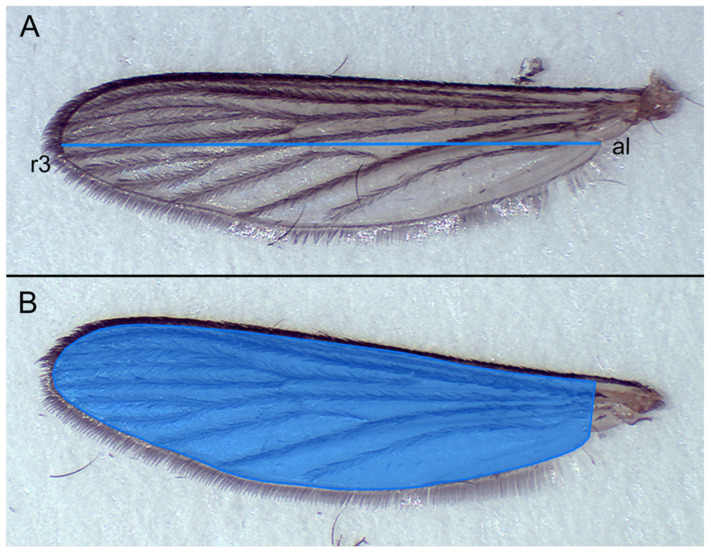
Measured wing traits. (**A**) Wing length was measured as the linear distance from the distal end of the alula (al) to the tip of the third radial vein (r3) at the apical margin. (**B**) Wing area was measured as the edge of the wing membrane and was limited to proximal size by a straight line forming a 90° angle with the wing rib and connecting it to the posterior edge of the alula.

Although we could not detect CYV in the offspring, we still considered the question of whether CYV infection of the parental generation might have an impact on the development of the offspring. For this purpose, we measured the wings of the offspring of the CYV-infected mosquitoes. Examination of the morphological characteristics of the virus-affected (CYV group) and non-virus-affected (control group) progeny revealed significant differences in terms of wing sizes, which were divided into wing length (WL) and wing area (WA) (Figure 6).

With a mean length of 3.453 mm (±0.1806), the WL of the female CYV group was significantly reduced compared to the female control group (3.507 mm (±0.1934)) (Table 2). Similarly, the mean WL of the male CYV group (2.893 mm (±0.1395)) was significantly smaller than that of the control group at 2.968 (±0.1445). The measurement of WA also showed significant differences between the groups, as the WA of the female F1 generation of the control group was 2.791 mm^2^ (±0.3236), whereas the females of the CYV group had a significantly smaller WA of 2.699 mm^2^ (±0.2837). Males also showed a significant difference in WA between groups. The measured WA in the male control group averaged 1.851 mm^2^ (±0.1715) and was, accordingly, larger than that of the CYV group with a wing area of 1.766 mm^2^ (±0.1677) (Table 3).

### 3.6. Host Specificity

#### 3.6.1. In Vitro CYV Infection of Mammalian Cells

**Figure 7:**
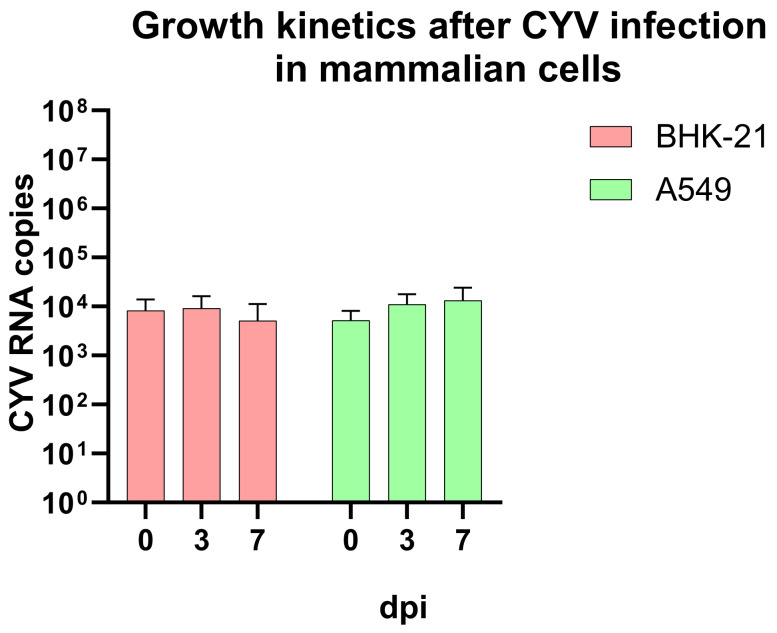
Viral growth kinetics of CYV in different mammalian cell lines. BHK-21 (red) and A549 (green) cells were inoculated with an infection volume of 2.5 µL of CYV displayed with SD. Samples were taken on days zero, three, and seven; dpi = days post-infection.

Furthermore, in our study, we wanted to confirm that CYV does not replicate in mammalian cells. Therefore, we inoculated BHK-21 and A459 cells with CYV and examined the cell culture supernatants at days zero, three, and seven. We could not observe CPE and, therefore, measured the RNA copies via qRT-PCR with our CYV standard. The inoculum with the infection volume of 2.5 µL in the mammalian cell infection experiments contained approximately 1.82 × 10^7^ viral RNA copies. For the BHK-21 cells, a decrease from the day of infection with a mean of 8.31 × 10^3^ (±4.80 × 10^3^) RNA copies to 6.09 × 10^3^ (±4.50 × 10^3^) RNA copies on the seventh day was observed (Figure 7). The results regarding the A549 cells showed a mean viral RNA with 5.25 × 10^3^ (±2.45 × 10^3^) RNA copies at day zero to 1.02 × 10^4^ (±9.35 × 10^4^) RNA copies at day seven in the A549 cells. We observed high variances in these data, with RNA copies ranging from 7.41 × 10^2^ to 2.43 × 10^4^ in the A549 cells at day seven. Taken together, and with regard to the high variances we observed in this experiment, we do not assume that CYV would replicate in mammalian cells.

#### 3.6.2. In Vivo Growth Kinetics of CYV in Other Insects

**Figure 8:**
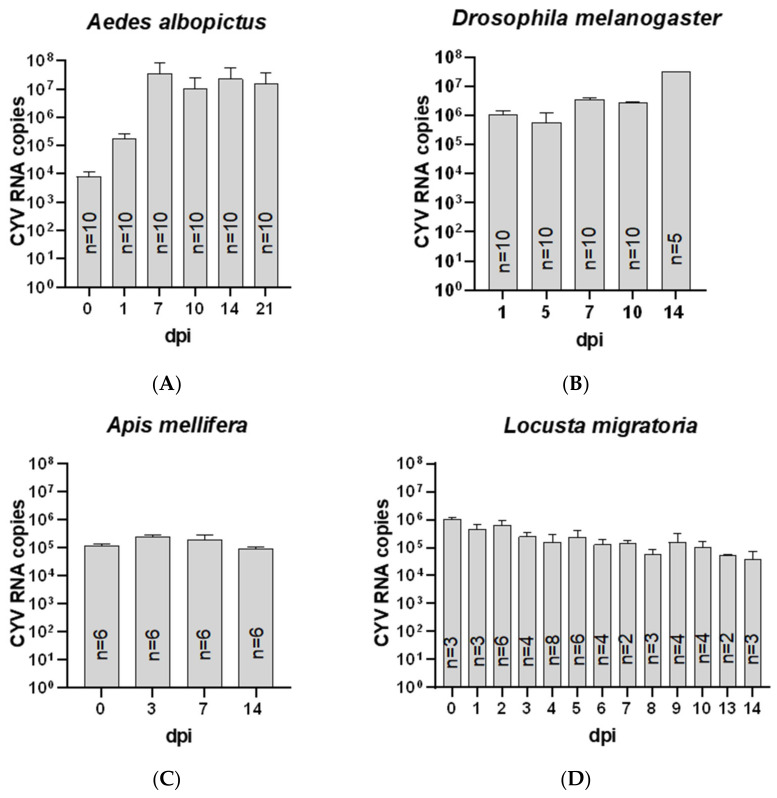
Viral growth kinetics in different insect species. Mean RNA copy numbers of all conducted replicates with SD are shown. (**A**) Growth kinetics in *Ae. albopictus* measured in pools with five males and five females per pool; (**B**) growth kinetics in *D. melanogaster* measured in pools with five males and five females per pool and sample day one, five, seven, and ten, and at 14 dpi only for the females; (**C**) growth kinetics in *A. mellifera* tested in pools of two bees per dpi; (**D**) growth kinetics in *L. migratoria.* Locusts were tested individually; dpi = days post-infection.

The injection of CYV into other insect species showed that the virus replicated in *Ae. albopictus*, with the highest RNA copy number on day five at 4.10 × 10^7^ (Figure 8A), and in *D. melanogaster*, with a peak of 6.74 × 10^6^ RNA copies on day fourteen (Figure 8B). RNA copy numbers in these species increased by up to three to four log levels over a 14-day observation period. In *A. mellifera*, the highest RNA copies were measured on days zero and three after the injection of CYV, with a mean copy number of 1.75 × 10^5^ (Figure 8C). On subsequent sample days seven and fourteen, RNA copies decreased to 8.86 × 10^4^ on day fourteen. In *L. migratoria*, the viral RNA copy number decreased over time, with the highest viral RNA copy number on the day of injection of 1.05 × 10^6^ RNA copies to day 14 post-injection with the lowest copy number of 3.72 × 10^4^ copies (Figure 8D). Altogether, we have demonstrated that CYV replicates in *Ae. albopictus* and *D. melanogaster*, both belonging to the order Diptera. In the other insect species we tested, namely, the honey bees and the locusts, CYV replication could not be measured.

#### 3.6.3. Survival Rates

**Figure 9:**
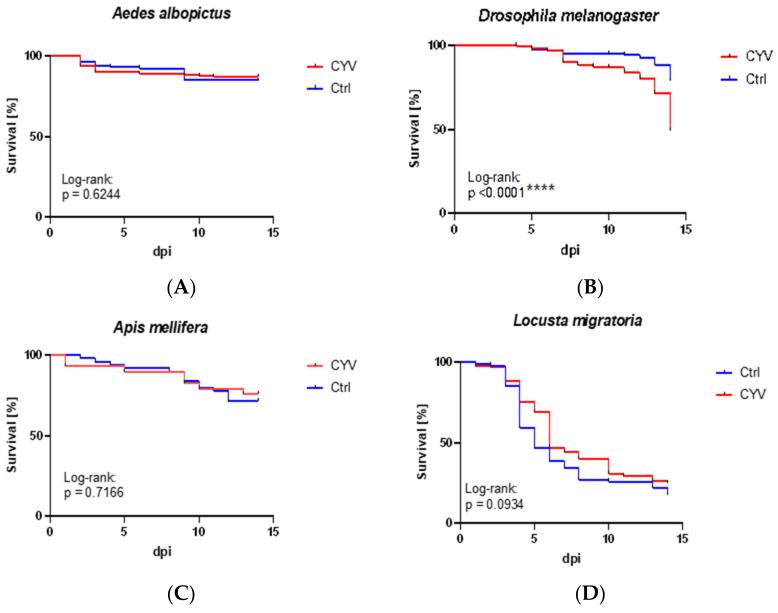
Survival curves. The data represent the survival rates in percentages over days post-infection (dpi). (**A**) *Ae. albobictus*: CYV-injected vs. medium-injected; Log-rank: *p* = 0.6244 (ns). (**B**) *D. melanogaster*: CYV-injected vs. medium-injected; Log-rank: *p* = <0.0001 (****). (**C**) *A. mellifera*: CYV-injected vs. medium-injected; Log-rank: *p* = 0.7166 (ns); two replicates. (**D**) *L. migratoria*: CYV-fed females vs. medium-fed females; Log-rank: *p* = 0.0797 (ns). Red curve—CYV-infected insects; blue curve—control (Ctrl) insects.

Next, we wanted to determine whether the CYV injection had any effect on the survival of *Ae. albopictus*, *D. melanogaster*, *A. mellifera,* or *L. migratoria*. Therefore, we observed the insects for 14 days, counted the surviving individuals, and evaluated these data at the end of the experiment using Kaplan–Meier curves. The survival rates showed no significant differences between the CYV-injected and control groups with respect to *Ae. albopictus* (log-rank: *p* = 0.6244) (Figure 9A). When comparing the survival rates of the males and females with the respective control groups, again, there were no significant differences in the survival rates (for females: log-rank: *p* = 0.1227 and for males: log-rank: *p* = 0.3828). The results of the survival tests performed on the fruit flies showed a significantly lower survival rate of the CYV-injected flies compared to the control flies (log-rank: *p* < 0.0001) starting from the ninth day of fly death (Figure 9B). In the honey bees, no significant differences were observed between the control and virus-injected groups, with a *p*-value of 0.7166 (Figure 9C). No significant differences in survival rates were observed in the locusts between the CYV-injected and control groups, with a *p*-value of 0.0934 (log-rank) (Figure 9D).

## 4. Discussion

Insect-specific viruses are found across a wide range of virus families, including virus families mainly known for their arbovirus representatives, such as the flaviviruses. Accordingly, most data on ISVs in mosquitoes have been obtained from these arbovirus-related ISVs, such as CFAV or CxFV. In the case of CxFV, the potential to suppress or at least delay arbovirus transmission when co-infected in cell culture with DENV and WNV was described. Furthermore, a lower rate of viral dissemination in *Ae. aegypti* mosquitoes was observed for ZIKV when co-infected with CFAV [13]. Similarly, the co-infection of WNV and the insect-specific flavivirus NHUV resulted in a reduced vector competence of mosquitoes with respect to WNV [34].

In contrast to CxFV and CFAV, less is known about CYV, which was discovered in 2010 in hibernating, free-living *Cx. pipiens* mosquitoes [17]. Following confirmation of its insect origin and phylogenetic classification in the genus Entomobirnaviruses [17], further studies investigated immunological responses to CYV infection in vitro [30,35]. Franzke et al. (2018) found that different cell cultures (Aag2, U4.4, and C7-10) are persistently infected with CYV and that infection rates vary across different passages [30]. Furthermore, the involvement of RNA interference (RNAi) in the control of CYV was confirmed through small RNA sequencing [30,35]. Similar to many other insect viruses, an inhibition of the RNAi pathway through a CYV protein was also confirmed [35]. Besides this information on immune interactions in mosquitoes, nothing is known regarding the transmission cycle of CYV nor the impact of CYV infection on mosquitoes. It is not even known if CYV is a *Culex* specific virus or if it can infect a wide range of insects.

We found that CYV was able to replicate in *Cx. pipiens* biotype *molestus* laboratory strains originating from the north of Germany after injection and oral uptake. However, the replication rate was significantly higher after injection (Figure 1 and Figure 4) since this infection route bypasses two important tissue barriers: the midgut infection barrier and the midgut escape barrier [36,37]. Bypassing those barriers, a systemic infection with high RNA copy numbers for CYV was established in all the tested organs. The rapid spread of CYV after injection may be due to its distribution in the mosquito body via the hemocoel. Fu et al. (1999) showed that the spread of bluetongue virus (BTV) after intrathoracic injection in *Culicoides variipennis* begins rapidly and reaches an average RNA copy number after three days [38]. Our data showed a comparable pattern without the eclipse phase or partial eclipse phase that has been described for viral dissemination after oral ingestion [39]. This phenomenon describes a decrease in viral particles within the first two days after the ingestion of a viremic blood meal in which no or at least a few viruses are detected [39]. After the oral ingestion of CYV, such an eclipse phase was observed in *Cx. pipiens* biotype *molestus*. In total, we observed reduced replication of CYV, which resulted in lower RNA copy numbers as compared to the injection. However, after an initial loss of viral RNA copies due to the digestion of the inoculum, we observed an increase in viral RNA copies in all tested organs except the salivary glands (Figure 5). The absence of replication in the salivary glands might be due to the delayed replication of CYV after oral infection. For many organs, the highest RNA copy numbers were measured at fourteen days post-infection, whereas the injection of the virus led to high viral RNA copy numbers already three and seven days after infection. In contrast to our findings, oral infection with PCV in *Cx. annulirostris* failed, whereas intrathoracic injection led to 100% infection rates [40]. For arboviruses, the dissemination of virus particles in mosquitoes after oral ingestion is well-described. Only a small number of midgut epithelial cells are thought to be susceptible to initial viral infection [36]. For ISVs, and especially for CYV, the pattern of infection in the mosquito body has not yet been described, but an initial infection of only a few cells could explain the decrease in viral RNA measured in our data on the third day after oral infection. We do not see a sex-specific dissemination pattern of CYV after feeding or injection. This result, both for viral growth in vivo after injection and after oral infection, seems logical when one considers that both males and females are constantly exposed to different ISVs in nature. When we compare this pattern, we suggest that the barriers CYV must overcome are similar to those described for arboviruses. When examining the RNA copies measured within one hour after feeding with CYV, we already found the virus in all the tested organs. This phenomenon has been observed in earlier studies in *Ae. aegypti* with Uganda S, yellow fever virus (YFV), and Semliki Forest virus and in *Ae. australis* with Whataroa virus [41,42]. Virus was detected in the hemocoel shortly after the mosquitoes fed on a viremic blood meal and, therefore, spread throughout the mosquitoes’ bodies. It was hypothesized that viral particles could enter the hemolymph via an intracellular route, which could be described as a leak [43]. This so-called “leaky gut phenomenon” could be a possible explanation for the rapid distribution of CYV after feeding in our experiments. In addition, the contamination of the different organs during the process of dissection despite careful washing could account for the measured RNA copy numbers. Similar to mammals dissected without perfusion prior to dissection, contamination with a few viral particles via body fluids could account for a false-positive organ sample. False-positive organ samples can only be ruled out by the staining of the tissues with a virus specific antibody. However, such a staining procedure would only be informative at later time points of infection, as no viral protein production can be expected directly following injection.

It can be hypothesized that one possible route of CYV transmission within mosquito populations is indirect transmission via virus-contaminated food sources, as we have shown that CYV uptake by *Cx. pipiens* biotype *molestus* leads to infection. Furthermore, we were able to demonstrate that CYV is secreted in the saliva, as all the tested saliva samples were positive for CYV after injection experiments, although the measured RNA copy numbers were low. Considering that mosquitoes salivate while feeding [44], the virus could be regurgitated onto food sources and ingested by the following mosquitoes. A similar infection route via sugar meal sources has already been described for Massilia virus, a sandfly-transmitted arbovirus, in *Phlebotomus perniciosus* [45], and experiments with *Cx. annulirostris* and *Cx. gelidus* have shown that, following infection with various flaviviruses, the virus can be delivered to sucrose food sources, albeit in varying amounts depending on the species and virus [46]. Since ISVs are unable to replicate in vertebrate cells, one possible route of transmission could be via plant food sources. However, further experiments are needed to clarify this, since we do not know if the secreted amounts of CYV are sufficient to induce infection in naïve mosquitoes

Besides contaminated plant food sources, vertical transmission if often described as a primary transmission route of ISVs. Specifically, baculoviruses and polydnaviruses rely on this mode of transmission; additionally, RNA viruses such as Sigma virus in *Drosophila* utilize this mode of transmission [47]. Regarding mosquito-associated ISVs, very little information on vertical transmission is available and solid evidence for this form of transmission only exists for three viruses: CFAV, NHUV, and Kamiti River virus (KRV). In contrast, vertical transmission is described for many arboviruses in mosquitoes (SINV, RVFV, and ZIKV). This strategy ensures viral survival even in periods between disease outbreaks, e.g., in dry seasons, when adult mosquitoes are absent [10,48,49,50,51,52,53].

For CYV, however, we were unable to demonstrate vertical transmission, although we were able to show that the virus spreads to the reproductive organs and replicates there in male and female *Cx. pipiens* biotype *molestus* mosquitoes (Figure 1). This was also described for PCV, where no vertical transmission occurred after intrathoracic injection of the virus [54]. Possible explanations for the lack of vertical transmission in our experiments could be due to the use of inbred laboratory mosquito strains or the virus exposure schemes. Another possibility could be that vertical transmission does not occur until later gonotrophic cycles; thus, the examination of the offspring of autogenous eggs laid within the first five days could be too early [55]. Anderson et al. (2008) found that the vertical transmission of WNV (after oral infection) in *Cx. pipiens* biotype *pipiens* mosquitoes does not occur until the thirteenth day after infection [56]. The results of Saiyasombat et al. (2011) also showed no vertical transmission of CxFV after the inoculation of uninfected *Cx. pipiens* biotype *molestus* mosquitoes colonized in the laboratory [50]. However, they showed that CxFV-positive females collected in the wild transmitted the virus vertically, with a prevalence of 100%. Intrathoracic injection is likely to play a minor role in preventing vertical transmission. This assumption is based on the already-successful demonstration of vertical transmission after intrathoracic injection for various bunyaviruses [57,58], ZIKV [28], YFV [59], or Japanese encephalitis virus (JEV) [60]. Vertical transmission appears to be the most obvious explanation for the prevalence of ISVs within a mosquito population, which is mainly suggested when males and females are both naturally infected [51].

Since vertical transmission does not seem to be an option for CYV, we also tested whether the virus could be transmitted horizontally by venereal transmission. The virus occurs in nature and is not an artifact of cell culture in the laboratory such as the closely related Espirito Santo virus and Drosophila X virus. Both belong to the *Birnaviridae* family but have never been found in nature in mosquitoes or fruit flies [61,62]. For CYV, we found clear evidence that the virus can survive in a mosquito population by being transmitted between the sexes. Venereal transmission has been frequently described for arboviruses (SINV, St. Louis encephalitis virus (SLEV), or ZIKV), but it has also been described for ISVs, such as Deformed Wing Virus (DWV) in honey bees, which was additionally transmitted vertically after the infection of a queen bee with DWV-positive sperm, and it has also been described for CFAV and Aedes Flavivirus (AEFV), two ISVs belonging to the *Flaviviridae* [63,64,65,66,67,68]. For the above examples, namely, SINV, SLEV, ZIKV, DWV, CFAV, and AEFV, all of these viruses can also be transmitted vertically within an insect population [48,66,67,68,69,70]. This contrasts with our results for CYV, wherein we found no evidence of vertical transmission, as mentioned earlier. We cannot exclude the possibility that the vertical transmission of CYV occurs naturally and that our results represent the effects of inbred mosquito strains and laboratory conditions. Apart from this, as well as the fact that we could not find another example of a virus in the literature that enables venereal but not vertical transmission, there is a need for more research in this area.

Next, the impact of a CYV infection on mosquitoes and their survival and fertility was investigated. We detected a significantly higher mortality rate for *Cx. pipiens* biotype *molestus* males in contrast to females. A selective male-killing phenomenon is already known as a possible strategy for controlling mosquito populations and has already been reported for insects via infection with bacteria, e.g., with *Wolbachia, Rickettsia*, *Spiroplasma*, *Flavobacteria*, or *Arsenophonus,* or microsporidia [71,72,73,74]. Viral male-killers were analyzed for the insect *Homona magnanima* and the oriental tea tortrix, which can be infected by Osugoroshi viruses (OGVs) [75]. CYV infection could potentially be used as a biological agent to kill males and subsequently reduce mosquito population size. In terms of vector control, this higher mortality of males could be of interest considering, on the one hand, the reduction in mosquito populations and, on the other hand, the high reproductive rate of males, who can mate and spread their genetic material for the entirety of a mosquito’s life [76]. This contrasts with females, who are limited to oviposition in order to pass on their own genetic material. Furthermore, we could not find any impact on the fertility of mosquitoes if they had mated before CYV infection, although the reproductive organs are rapidly flooded and infected by the virus. The amount of F1 larvae hatched from the laid eggs as well as the pupation rate or the growth to adult mosquitoes of the offspring of the CYV-infected mosquitoes showed no significant differences compared to the control mosquitoes. Furthermore, the sex ratio—more specifically, the female ratio—of the adult F1 generation was determined. Influencing this trait, e.g., by a male bias, would affect the reproductive ability of the F1 generation. However, no significant change in the female proportion of the F1 generation was detected. There are, however, other possible traits influenced by CYV that could have an effect on the reproductive success of the offspring in terms of morphological traits, which we investigated using wing sizes. Our results show that the wing sizes of the female and male offspring of the CYV-infected mosquitoes were significantly reduced (Figure 6, Table 3). Such a decrease in wing size might suggest a decline in the fecundity of the F1 generation [77,78,79]; thus, the application of CYV in the parental generation might have had an impact on the reproductive success of the following generations. Since we were unable to demonstrate transovarial transmission, these changes might be due to epigenetic or immunomodulatory factors in infected ovaries. Epigenetic effects have been described in plants, mammals, and insects [80,81,82]. These effects include both behavioral and physiological traits that can be influenced by environmental changes or pathogenic infections and transmitted from the parental generation to the offspring [82]. In insects, phenotypic variation, transgenerational immune priming, declining insecticidal sensitivity, and the effects of diet on the offspring generation triggered by various influences on the parental generation, among others, have been described. [83,84,85]. We hypothesize that a CYV infection of the parental generation could affect the offspring generation, even if no transovarial transmission occurs. These effects may be phenotypic, such as the smaller wing sizes described herein, or non-phenotypic. However, further studies are needed to validate the notion that this effect negatively impacts offspring and whether non-phenotypic effects, such as immune priming or influences on offspring fertility, are also detectable by CYV infection of the parental generation. Affecting the reproductive output of mosquito colonies could promote population reduction, ultimately leading to lower population sizes.

Finally, we analyzed the host specificity of CYV. If CYV is to be used as a tool to reduce mosquito population size or to interfere with arbovirus transmission, the environmental safety of this agent needs to be confirmed to prevent unwanted impacts of the vector control measurements on other insects. We were able to confirm that the virus could not replicate in any mammalian cell culture. This finding emphasizes the assumption that CYV may be insect-specific. By examining closely and distantly related insect species, we found that CYV is not mosquito-specific but possibly diptera-specific; *Ae. albopictus*, a second tested mosquito species, seems to be unaffected by CYV, while the closely related *Drosophila* (also order Diptera) is affected by this virus (reduced survival Figure 9B). To determine whether other insect orders might be affected, we added an economically important species from the Hymenoptera order, the honey bee, and a member of the Orthoptera order, the economically relevant agricultural pest insect *L. migratoria*. The survival rates of both the honey bee and the locust were not affected by CYV infection (Figure 9C,D), as confirmed by a lack of replication of the virus (Figure 8C,D). These results suggest that CYV may be specific to Nematocera and Brachycera but does not always exhibit pathogenic properties in these species. However, this needs to be further validated for these and other species of these two suborders.

Taken together, due to the male-killing effect and the reduced wing size, which might be associated with the reduced reproductive success of subsequent generations, CYV may be considered for use as a tool to destabilize arbovirus vector populations. Regarding the safety of the CYV agents, we were able to show that no harmful effects on economically important organisms such as honey bees could be observed.

## Figures and Tables

**Table 1:** Summary of statistical analysis of viral growth in male and female carcasses. Multiple *t*-tests with Bonferroni correction were performed to determine *p*-values; below, the *p*-values are shown for carcasses of both sexes compared between all measured time points tested.

	Carcass					
	0 vs. 3 dpi	0 vs. 7 dpi	0 vs. 14 dpi	3 vs. 7 dpi	3 vs. 14 dpi	7 vs. 14 dpi
Males	0.0141	0.0177	0.0086	ns	ns	ns
Females	ns	0.0425	ns	ns	ns	ns

**Table 2:** Summary of various reproductive traits comparing CYV- and medium-injected *Cx. pipiens* biotype *molestus* females with SD. For parametric data, the unpaired *t*-test was performed to determine *p*-values; *p*-values of non-parametric data were determined using the Mann–Whitney test.

Traits	CYV	Medium	*p*-Value
Egg rafts per female	0.66 (±0.157)	0.59 (±0.121)	0.4597
Eggs per female	26.3 (±10.48)	21.4 (±9.97)	0.4972
Eggs per raft	39.2 (±10.23)	35.6 (±9.73)	0.6088
Egg hatching rate (%)	76.9 (±9.13)	88.2 (±9.84)	0.0982
Pupation rate (%)	88.7 (±11.48)	97.3 (±3.79)	0.0864
Emergence (%)	82.8 (±16.15)	96.0 (±5.39)	0.1000
Percentage of females (F1) (%)	49.3 (±6.34)	55.4 (±4.15)	0.1558

**Table 3:** CYV induced alteration in the morphological trait wing size. Change in wing length (WL) and wing area (WA) with SD was defined by determining the difference for both traits between the offspring of CYV-injected females and the offspring of medium-injected females. All *p*-values are based on the Mann–Whitney test for nonparametric data. Sample size: CYV female: n = 295, CYV male: n = 286, control female: n = 102, and control male: n = 97.

	CYV		Control		*p*-Value	
	WL [mm]	WA [mm^2^]	WL [mm]	WA [mm^2^]	WL	WA
Females SD	3.453 ±0.1806	2.699 ±0.2837	3.507 ±0.1934	2.791 ±0.3236	0.0298	0.0162
Males SD	2.893 ±0.1395	1.766 ±0.1677	2.968 ±0.1445	1.851 ±0.1715	<0.0001	<0.0001

## Data Availability

Not applicable.
